# Factors influencing changes in medication-taking and driving behavior after warnings about prescription medications that prohibit driving: an online survey

**DOI:** 10.1186/s12889-022-13407-2

**Published:** 2022-05-21

**Authors:** Yasue Fukuda, Moemi Saito

**Affiliations:** 1 Facility of Pharmaceutical Science, Suzuka University of Medical Science, 3500-3 Minami-tamagaki, Suzuka, Mie 513-8670 Japan; 2 Faculty of Pharmaceutical Sciences, Tokyo University of Pharmacy and Life Sciences, 192-0392 Horinouti Hachioji city, Tokyo, Japan

**Keywords:** Medication warnings, Driving behavior, Health literacy, Adherence, Social environment

## Abstract

**Background:**

This study examined warning messages as a strategy for preventing automobile crashes by drivers on medications. We investigated the degree of awareness regarding the effects of medication on automobile driving and changes in medication-taking and driving behavior. We also assessed associations between socio-environmental factors and the driving and medication-taking behavior adopted by individuals after being warned about driving-related risks.

**Methods:**

Responses to an online questionnaire from 1200 people with a driving license who were taking prescription medications at the time of inquiry (March 2019) were collected and analyzed. The items surveyed were sex, age, educational history, health literacy, current medications, and medication-taking and driving behavior after being warned.

**Results:**

Of the total respondents, 30% were taking medicine that prohibited driving. Of those taking prohibited medications, 25.7% did not receive a warning about driving from healthcare professionals. Most respondents taking prohibited medications received euphemistic warnings, such as “practice caution” (30%), “refrain from calling attention” (29.4%), and “avoid driving” (19.8%); 16% of the direct warnings were about not driving. Medication’s effects on driving were recognized by 80% of the total respondents. The degree of awareness was significantly higher among respondents taking medications that prohibit driving than among those taking medications that did not prohibit driving or those taking unknown medications. Awareness of medicine’s influence on driving was associated with health literacy. No association was found between age, gender, health literacy, history of side effects, and driving and medication-taking behavior. Approximately 22% of respondents adjusted their medication use at their discretion and 39% maintained treatment compliance but continued driving. Among respondents taking medications that prohibit driving, whether driving was required for work was a significant factor in their driving and medication-taking behavior after being warned.

**Conclusions:**

Healthcare professionals do not always fully inform patients about the driving-related risks of medications. To encourage patients who are taking medications that have a significant impact on their driving to either stop driving or consult a healthcare professional, healthcare professionals must first understand the patient’s social environment, such as whether driving is required for work, and then create an environment conducive to advice-seeking.

## Background

It has been reported that the use of prescription medicines is associated with traffic accidents [1, 2]. Driving an automobile requires cognitive, motor, and sensory functions that encompass visual and auditory senses [3, 4]. Some prescription medications, such as psychoactive medicines, can affect the visual field and visual perception and cause loss of consciousness, while others can affect cognitive and motor functions [5, 6]. For example, antidepressants and sedatives affect the neural system, leading to drowsiness and reduced cognitive function [7]. It has also been pointed out that hypoglycemic agents may result in symptoms of hypoglycemia and decreased consciousness [8]. Consequently, numerous countries have car-driving regulations for individual medicines depending on their impact on driving behavior [9, 10]. According to a European study, medicines are classified into four types: banned medicines that have a significant impact on driving, medicines with moderate impact, medicines with little impact, and medicines that have no impact [11]. Meanwhile, in Japan, medicine package inserts provide information on the medicine’s efficacy, as well as a warning about side effects, which are classified into three categories for driving impairing medication [12]. The first category includes medicines that can significantly hinder car driving and for which package inserts state that care should be taken not to drive a car or operate machinery while taking such medicine. Healthcare professionals prohibit driving after patients have taken such medicines. The second category includes medicines that require attention, depending on the patient’s condition, and a warning against driving a car or operating a machine is included. The third category includes medicines that do not come with a warning about driving a car. The consequence of taking medicines that hinder driving or require attention is severe when injury or loss of life is involved. Therefore, Article 3 of the Act on Punishment of Acts Inflicting Death or Injury on Others by Driving a Motor Vehicle which was established in May 2014 provides as follows:

“A person who drives a motor vehicle in a state likely to hinder safe driving under the influence of alcohol or medications, and thereby comes to have difficulty in driving safely under the influence of such alcohol or medications, is subject to punishment by imprisonment with work for not more than 12 years when the person thereby causes injury of another; or imprisonment with work for not more than 15 years when the person thereby causes death of another” [13].

In 2020, a car-driving punishment law was applied to an older adult in Japan who injured and killed two female students due to loss of consciousness after taking a prostate treatment medicine [14]. There are similar legislations related to medications’ impact on driving in the United States and Europe [15, 16].

The spread of infectious diseases, such as COVID-19, has hastened the trend away from public transportation and toward private automobiles, increasing the need to consider the effects of medication on driving [17, 18]. Healthcare professionals must ensure that patients fully understand the risks of medications before continuing them. Patient awareness, knowledge, and directionality regarding the risks of medication are reportedly associated with the age and education level of car drivers [19]. However, from a risk-management perspective, researchers do not fully understand the degree of patient awareness regarding the effects of prescribed medications on driving or the influence of this awareness on driving and medication-taking behavior.

Car drivers must understand the effects of medications on their driving ability and prevent accidents by taking appropriate action based on the information on side effects. Information provided by healthcare professionals likely influences not only driving behavior but also adherence to prescription medication. Thus, more research on this topic is necessary to enable healthcare professionals to provide adequate medication counseling. Moreover, healthcare professionals should be able to understand how people behave in order to encourage behavior change through warning messages.

Verster et al. indicate that drivers find it difficult to objectively predict and evaluate their own driving performance [20]. Further, a driver’s behavioral intention might not always match their actual behavior. It is possible to avoid the risks associated with driving while on medication by predicting the relationship between relevant information and warning messages provided by healthcare professionals and drivers’ behavioral characteristics. Medication management interventions tailored to individual patients can also be applied.

Studies on drunk driving and truck driving behavior have shown that behavioral intent and attitude are predictors of driving behavior. Aizen’s theory of planned behavior has been applied in research on the relationship between drinking and driving and the driving behavior of truck drivers in terms of risk aversion and behavior control of automobile accidents [21, 22].

**Fig. 1:**
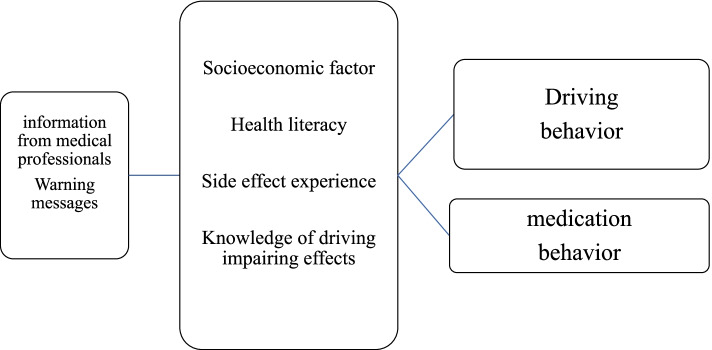
Warning message and driving behavior, adherence process

In this study, we sought to elucidate the predictors of and relationship between patients’ medication-taking behavior and intentions to engage in safe car-driving behavior by providing driving information for prescribed medicines, based on Monteiro et al.’s research shown in Fig. 1 [19].

The objective of this study was to elucidate, from a risk-communication perspective, how demographic factors influence patients’ awareness regarding the effects of medications that prohibit driving and how they influence patients’ driving and medication-taking behavior after receiving appropriate warnings.

## Methods

### Survey design and participants

This was a cross-sectional survey study conducted via online platform. We conducted a questionnaire survey from March 15 to 30, 2019, on 1300 citizens who were taking medications and were enrolled in Cross Marketing Inc., an online research company and survey provider. From the information gathered by Cross Marketing Inc., those who were prescribed medicine at the hospital within the last year were extracted and requested to be investigated. The pretest of the Internet survey was conducted from March 9 to 14, 2019, and the main survey was conducted from March 15 to 30, 2019. To exclude respondents who did not read the questions, a question asking respondents to select the first answer from the five options was included. Respondents who did not select the first option were excluded.

A total of 9854 citizens were initially screened as potential participants. Of them, 2700 citizens responded to the main survey. Finally, 1300 responses were obtained by excluding the answers of those who did not have a driver’s license and whose age or gender was inconsistent with the registered gender or age. One hundred responses with an answer time of less than 3% were also excluded. After applying the exclusion criteria, data of 1200 respondents were retained for analysis. Participants comprised adult men and women aged 20–79 years with an automobile driving license and on medications prescribed by a medical institution.

### Survey items

Questions were asked regarding eight topics, based on previous studies [15, 19, 23]. The topics included respondent attributes (e.g., gender age), educational history (junior high school graduate, high school graduate, junior college, university, or higher graduate), health literacy (based on Suka’s Health Literacy Scale [24]), driving required for work (yes/no), history of side effects (yes/no), and experience of side effects such as drowsiness and lightheadedness. Participants were also asked about the prescription medicine they were currently taking and the researchers verified and analyzed this list. Participants were questioned on their degree of awareness regarding the effects of medication on driving, and responses were given on a five-point Likert scale (1 = not at all, 2 = only a little, 3 = to some extent, 4 = rather much, and 5 = very much). Finally, medication names entered by respondents were checked by researchers and classified according to their therapeutic effect; medications that prohibit driving based on Japanese medication package inserts were extracted [25]. Respondents were grouped into three—those taking medications that prohibited driving, those taking medications that did not prohibit driving, and those unaware of what medication they were taking. The degree of awareness regarding the effects of medication on driving was compared between the groups. Health literacy and attributes, such as gender, age, and driving frequency, were also analyzed to determine their relationship with driving and medication-taking behavior after receiving a warning in this regard from healthcare providers.

### Statistical analysis

Data on respondents’ attributes and their degree of awareness regarding driving were tabulated. A chi-square test was performed to analyze the relationship between respondents’ degree of awareness and the effects of medication on driving. We analyzed how being warned by a healthcare professional affected driving and medication-taking behavior among respondents taking medications that prohibited driving. Moreover, to minimize the omission of some medications, responses describing respondents’ medication-taking and driving behavior after being warned were grouped into negative and positive behavior types. Negative behaviors related to changes in taking medication made at the respondent’s discretion included “reduced the dose,” “took medication at longer intervals,” and “stopped the medication” but “continued driving” regardless of driving frequency. Positive behaviors included “took medication and stopped driving as instructed by physician or pharmacist” or “consulted a healthcare professional.” These objective variables were binarized by assigning “0″ to negative behaviors and “1″ to positive behaviors. Logistic regression analysis was performed to determine factors that affect medication-taking and driving behavior among respondents taking medications that prohibit driving, with medication-taking and driving behavior as objective variables and gender, age, educational history, health literacy, driving required at work (yes/no), and history of side effects as explanatory variables. A two-sided significance level of 5% was used in all tests. All statistical analyses were conducted using SPSS ver. 26 (SPSS Inc., Chicago, IL, USA).

### Ethical considerations

Personal identifying information were excluded from the online survey. Moreover, “do not want to answer” was included as an answer choice for questions regarding educational history and other personal information. A document explaining the objectives of the survey, how the information would be disclosed, the voluntary nature of the study, details about the questions, and contact information of the principal investigator, was published online, along with an informed consent document. The questionnaire survey was conducted only with respondents who provided informed consent.

## Results

### Respondent characteristics

Respondent characteristics are shown in Table 1. The mean age of the respondents was 49.45 (±16.41) years. There were 275 respondents (22.9%) with a history of medication side effects such as sleepiness, lightheadedness, and dizziness, and 925 respondents (77.1%) with no history of side effects. There were 207 respondents (17.3%) who drove for work and 993 respondents (82.7%) who did not.

**Table 1:** Participant characteristics (*N* = 1200)

Gender	Male	600 respondents (50%)
	Female	600 respondents (50%)
Age	49.45 ± 16.41 years	
Educational history	Junior high school graduate	22 respondents (18.3%)
	High school graduate	353 respondents (29.4%)
	Specialized high school/vocational school/junior college graduate	248 respondents (20.7%)
	University or higher graduate	577 respondents (48.1%)
History of side effects	Yes	275 respondents (22.9%)
	No	925 respondents (77.1%)
Driving required for work	Yes	207 respondents (17.3%)
	No	993 respondents (82.7%)

### Awareness regarding medications

When asked what medication(s) they were taking, 179 (14.9%) respondents answered, “Do not know.” The remaining 1021 respondents entered the name of the medication(s) they were taking.

### Groups listed according to medication impact on their driving

The risk when driving a car is high, and groups were classified into those who take medicines that prohibit driving (*n* = 370, 30.8%), those whose medications do not prohibit driving (*n* = 650, 54.2%), and those taking unknown medications or who gave inaccurate medicine descriptions (*n* = 180, 15%).

### Therapeutic classes of medications taken by all respondents

**Fig. 2:**
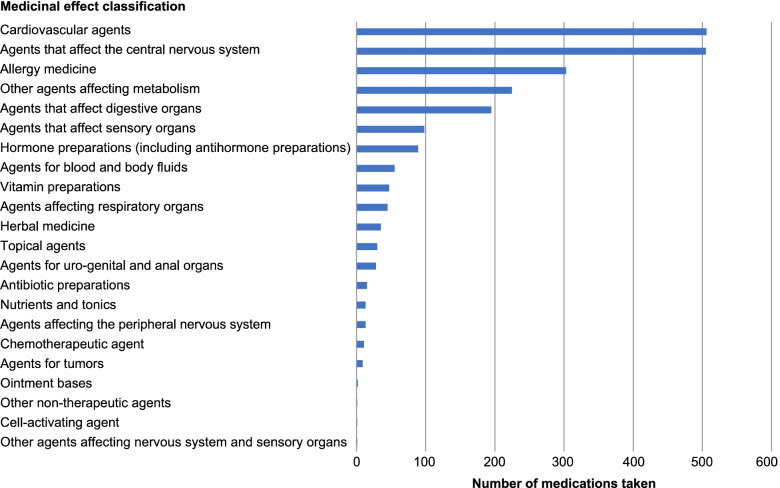
Medications taken by all participants (frequency classified by efficacy)

Medications were extracted from those being taken by all respondents. These medications are shown in Fig. 2, grouped by therapeutic class.

The most commonly taken medicines by respondents were for the cardiovascular system (*n* = 506) and the central nervous system (*n* = 505).

Classes of Medications Taken by Respondents that Prohibit Driving.

**Fig. 3:**
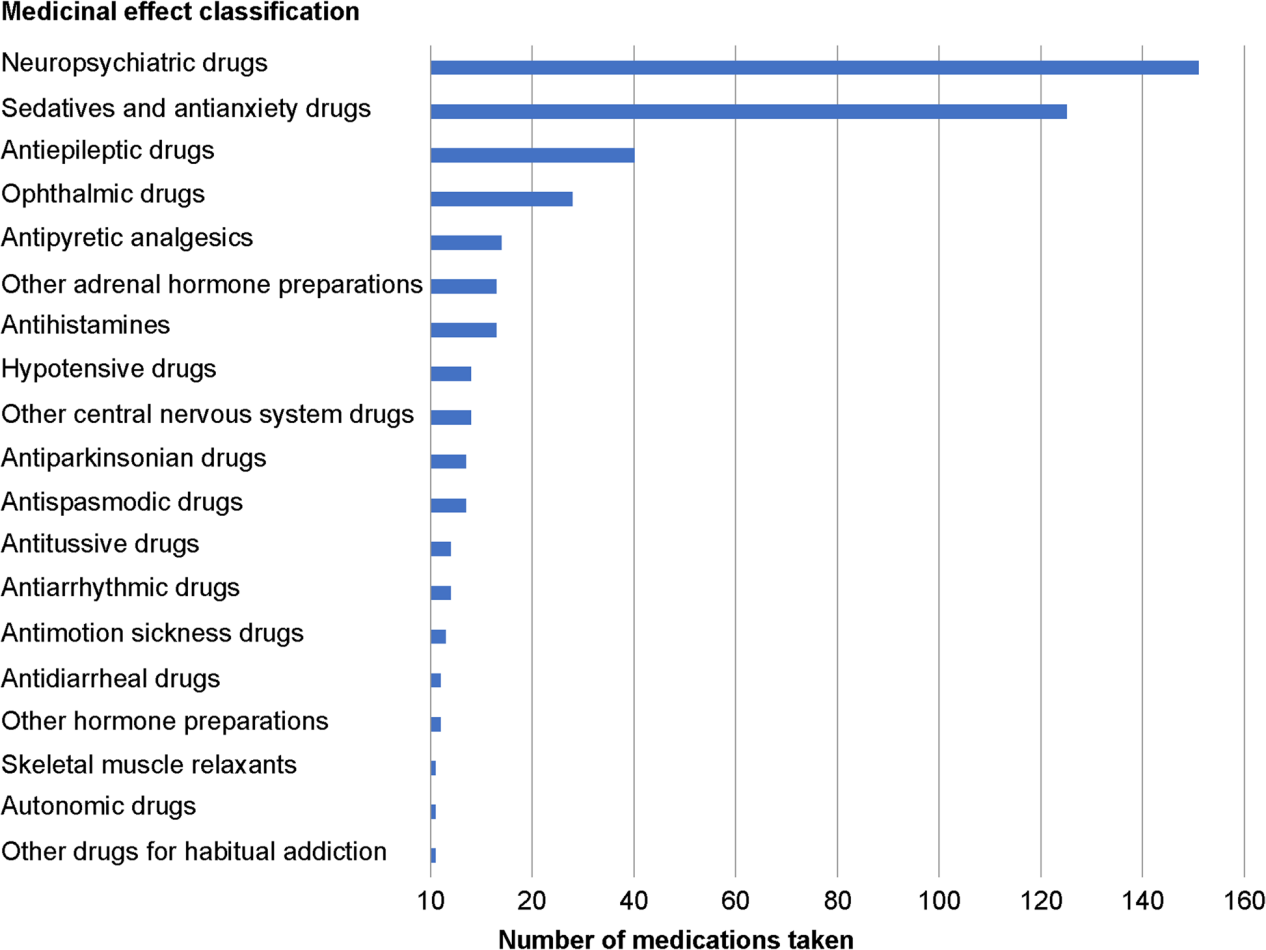
Therapeutic classes of medications taken by respondents that prohibit driving

Figure 3 shows the classification of medicines based on their high risk on driving and the frequency of medication.

The most common class was neuropsychiatric medications, which are prescribed for conditions such as schizophrenia (151 responses), followed by sedatives and anxiolytics (145 responses) and antiepileptic medications (40 responses).

### Awareness regarding effects of medication on driving

Participants’ awareness of the effects of medication on driving is shown in Table 2.

**Table 2:** Awareness of the effect of medication use on automobile driving

Response	Takes medications that prohibit driving		Does not take medications that prohibit driving		Takes unknown medications		Total
	*N*	%	*N*	%	*N*	%	*N*
1. Very aware	110	29.7%	116	17.8%	51	28.3%	277
2. Aware	209	56.5%	412	63.4%	82	45.6%	703
3. Neither aware nor unaware	29	7.8%	65	10.0%	24	13.3%	118
4. Not aware	20	5.4%	45	6.9%	17	9.4%	82
5. Not aware at all	2	0.5%	12	1.8%	6	3.3%	20
Total	370	99.9	650	99.9	180	100	1200

The effects of medication on driving were recognized by 81.8% of the respondents. The degree of awareness regarding the effects of medication on driving was significantly higher among respondents taking medications that prohibit driving than among respondents taking medications that did not prohibit driving or those taking unknown medications (*p* < 0.01). There was also a link between health literacy and awareness of the impact of medicines on driving impairment (*r* = 0.172; *p* < 0.01).

### Warnings provided by healthcare professionals to respondents taking medications that prohibit driving

Table 3 shows the content of warnings provided by healthcare professionals to respondents taking medications that prohibit driving. Among respondents taking medications that prohibit driving, 65.5% were warned about the effect on driving by a healthcare professional, 25.7% were not, and 8.8% did not know or could not recall whether they had received a warning. More than a quarter of the respondents stated that they are inexperienced or did not understand the associated risks and concerns.

**Table 3:** Warnings provided by healthcare professionals to respondents taking medications that prohibit driving (Multiple answers)

Warning	*N*	%
Do not drive while taking (using) this medication	50	16.0
Avoid driving while taking (using) this medication	62	19.8
Refrain from driving while taking (using) this medication It means to restrain oneself and stop it, but at the same time it can be interpreted to reduce the frequency	92	29.4
Practice caution during driving while taking (using) this medication	104	33.2
Other	5	1.6

Multiple responses were permitted because respondents may have used multiple pharmacies. The most common warning to prohibit driving was to “practice caution while driving,” which was received by 33.2% of respondents, followed by 29.4% who were warned to “refrain from driving” and 19.8% who were warned to “avoid driving”; as can be seen, these messages are euphemistic and rather unclear expressions. Meanwhile, only 16.0% were given direct messages not to drive.

### Respondents taking medications that prohibit driving: medication-taking and driving behavior after being warned

The medication-taking and driving behaviors of respondents taking medications that prohibit driving after being warned are shown in Table 4.

**Table 4:** Warnings regarding medication use and driving behavior among respondents taking medications that prohibit driving

	Group taking medications that prohibit driving	
	N	%
1.Stop taking (using) the medication/Drive at the same frequency as before	18	4.9%
2.Stop taking (using) the medication/Drive slightly less frequently	11	3.0%
3.Stop taking (using) the medication/Stop driving altogether	18	4.9%
4.Take (use) a lower dose of the medication/Drive at the same frequency as before	9	2.4%
5.Take (use) a lower dose of the medication/Drive less frequently	7	1.9%
6.Take (use) a lower dose of the medication/Stop driving altogether	0	0.0%
7.Take (use) the medication at longer intervals/Drive at the same frequency as before	10	2.7%
8.Take (use) the medication at longer intervals/Drive slightly less frequently	6	1.6%
9.Take (use) the medication at longer intervals/Stop driving altogether	3	0.8%
10.Take (use) the medication according to physician instructions/Drive at the same frequency as before	78	21.1%
11.Take (use) the medication according to physician instructions/Drive slightly less frequently	70	18.9%
12.Take (use) the medication according to physician instructions/Stop driving altogether	92	24.9%
13.Consult a physician, pharmacist, or nurse	41	11.1%
14.Adopt another approach/Other	7	1.9%
Total	370	100.1%

The results indicated that 22.0% of the respondents stopped taking the medication, reduced the dose, adjusted the frequency of the medication, or made some other adjustment at their discretion; moreover, 39.5% of respondents maintained treatment compliance according to physicians’ instructions but continued driving. In contrast, 25.2% of respondents took medication and stopped driving according to a physician’s instructions, and 11.4% consulted a physician, pharmacist, nurse, or another healthcare professional.

The relationship between the classification of the efficacy of prohibited medicines and medication driving behavior is shown in Table 5.

**Table 5:** Relationship between medical effect classification and medication-taking behavior and driving

Behavior	Medical effect classification													
	Neuropsychiatric medicines		Sedatives and antianxiety medicines		Antiepileptic medicines		Ophthalmic medicines		Antipyretics analgesics		Other adrenal hormone preparations		Antihistamines	
	n	%	n	%	n	%	n	%	n	%	n	%	n	%
1	6	4.0	3	2.1	0	0.0	2	7.1	0	0	2	15.4	2	15.4
2	4	2.6	7	4.8	2	5.0	0	0	1	7.1	0	0	1	7.7
3	6	4.0	7	4.8	3	7.5	4	14.3	0	0	0	0	0	0.0
4	5	3.3	5	3.4	0	0.0	0	0	0	0	1	7.7	1	7.7
5	5	3.3	5	3.4	0	0.0	1	3.6	0	0	0	0	0	0.0
6	0	0.0	0	0	0	0.0	0	0	0	0	0	0	0	0.0
7	8	5.3	8	5.5	2	5.0	0	0	0	0	0	0	1	7.7
8	5	3.3	5	3.4	0	0.0	0	0	1	7.1	0	0	0	0.0
9	0	0.0	3	2.1	0	0.0	1	3.6	0	0	0	0	0	0.0
10	34	22.5	26	17.9	4	10.0	0	0	4	28.6	4	30.8	3	23.1
11	24	15.9	20	13.8	12	30.0	8	28.6	5	35.7	1	7.7	1	7.7
12	35	23.2	37	25.5	11	27.5	7	25	2	14.3	5	38.5	3	23.1
13	14	9.3	17	11.7	5	12.5	4	14.3	1	7.1	0	0	1	7.7
14	5	3.3	2	1.4	1	2.5	1	3.6	0	0	0	0	0	0.0
Total	151	100.0	145	99.8	40	100.0	28	100	14	99.9	13	100	13	100

Driving and Medication-Taking Behavior

1. Stop taking (using) the medication/Drive at the same frequency as before; 2. Stop taking (using) the medication/Drive slightly less frequently; 3. Stop taking (using) the medication/Stop driving altogether; 4. Take (use) a lower dose of the medication/Drive at the same frequency as before 5. Take (use) a lower dose of the medication/Drive less frequently; 6. Take (use) a lower dose of the medication/Stop driving altogether; 7. Take (use) the medication at longer intervals/Drive at the same frequency as before 8. Take (use) the medication at longer intervals/Drive slightly less frequently; 9. Take (use) the medication at longer intervals/Stop driving altogether; 10. Take (use) the medication according to physician instructions/Drive at the same frequency as before; 11. Take (use) the medication according to physician instructions/Drive slightly less frequently; 12. Take (use) the medication according to physician instructions/Stop driving altogether; 13. Consult a physician, pharmacist, or nurse; 14. Adopt another approach/Other

Consultation with a healthcare professional, medication, and not driving according to physicians’ instructions were all defined as positive behaviors adopted after being warned, regardless of the type of prohibited medicines.

The data revealed the following. Fewer than half of the respondents were taking what they considered to be the correct response regardless of the type of prohibited medication. Antiepileptic drugs had the highest percentage of positive behaviors at 40%, while antipyretic analgesics and anti-inflammatory drugs had the lowest percentage of positive behaviors at 21.4%. Antihistamines had the highest rate of self-decisional discontinuation at 23.1%.

Dose adjustment was 0% for antiepileptic drugs and antipyretic analgesics. The highest rate of spacing of doses was 11% for hypnotic sedatives/anxiolytics. The rate of “take as directed by healthcare professionals but continue driving” was very high for antipyretic analgesics and anti-inflammatory drugs (64.3%). Almost the same number of respondents in the other drug groups as the response considered correct also indicated that they would take the medication as directed by the physician but would continue driving.

This could be because accidents caused by epilepsy patients have been reported and medical professionals are highly aware of the effects of car-driving accidents on patients with epileptic seizures.

This indicates poor communication of medicine-related risks by healthcare workers. In addition, there is a possibility that providing information on driving prohibition using ambiguous expressions from several health professionals may mislead patients’ judgments. It was shown that patients’ understanding of driving bans was inadequate and they often dealt with it on the basis of their judgment in most cases.

### Respondents taking medications that prohibit driving: analysis of factors affecting driving and medication-taking behavior (logistic regression analysis)

The results of the analysis of factors affecting driving and medication-taking behavior among respondents taking medications that prohibit driving (logistic regression analysis) are shown in Table 6.

**Table 6:** Factors affecting driving and medication-taking behavior of respondents taking medications that prohibit driving

	Coefficient	Standard error	*p*-value	Odds ratio	EXP (B) 95% confidence interval	
					Lower limit	Upper limit
Side effects (yes, no [2 categories])	0.126	0.246	0.61	1.134	0.7	1.837
Driving for work (yes, no [2 categories])	−1.104	0.361	0.002**	0.332	0.164	0.672
Health literacy	0.013	0.017	0.44	1.014	0.979	1.049
Junior high school education (reference)						
High school education	1.779	1.219	0.144	5.925	0.544	64.579
Vocational school/junior college education	0.114	0.263	0.667	1.12	0.668	1.878
University or higher education	−0.045	0.323	0.888	0.956	0.507	1.8
Gender (male = reference)	0.129	0.243	0.596	1.137	0.707	1.83
Age 20–29 years (reference)						
Age 30–39 years	0.271	0.437	0.536	1.311	0.556	3.089
Age 40–49 years	0.439	0.43	0.307	1.551	0.668	3.601
Age 50–59 years	0.064	0.431	0.882	1.066	0.458	2.48
Age 60–69 years	0.75	0.434	0.084	2.118	0.904	4.962
Age 70–79 years	0.254	0.469	0.589	1.289	0.514	3.232

**p < 0.01

Our analysis of the respondents’ positive behaviors revealed no association with age, sex, history of side effects, health literacy, educational history, or number of medications. There was a significant association between negative behavior and the need to drive for work.

Further, participants explained the reasons for continuing to drive after taking medications despite being warned. First, they believed that despite being drowsy, they would not cause an accident. Second, they were unaware of the extent to which the medicines they are taking affect their ability to drive.

## Discussion

This study examined from a risk-communication perspective, how demographic factors influence patients’ awareness regarding the effects of medications that prohibit driving and how they influence patients’ driving and medication-taking behavior after receiving appropriate warnings. According to the health belief model, people must understand that unsafe behavior increases the likelihood of an crashes and that crashes have a significant impact on one’s life and that of others. Patients must understand the benefits of safe behavior and think about what the obstacles to safe behavior are [26].

The results of this study suggest that healthcare professionals may not be rigorously warning patients. A previous study showed that the degree of risk awareness among patients is affected by the messages and expressions used by healthcare professionals. Phrases that were perceived as indicating the highest risk of automobile accidents were “please do not drive,” followed by “please avoid” and “please refrain”; this suggests that the details and expressions used while briefing patients about driving-related risks affected their perception of the level of risk [27]. The results of our study indicate that warnings are expressed differently by various healthcare professionals and that the warning expressions are not standardized.

This study also suggests that patients may not fully recognize the driving-associated risks of medications due to the indirect phrasing used when clinicians provide warnings to patients. Compared with illicit medicines, lawful medications are assessed as having a smaller effect on driving, which makes patients less disposed to refrain from driving when taking lawful medications [28]. Healthcare professionals should understand the risk classification of medicines and select appropriate alert messages for the inserts accordingly. Our findings suggest that ambiguous and euphemistic messages may not adequately convey the degree of risk to patients.

Our study revealed that respondents were aware that their medications had an impact on driving; however, 25% of the respondents continued to drive even when they were warned about the risks of driving while on such medication. Approximately 39% of respondents said they had high medication compliance, but it became clear that they were willing to continue driving. There was no association between medication and driving behavior, and participants’ health literacy, gender, and educational background; there was, however, an association with the presence of occupational driving necessity. Taking medications that affect automobile driving is an unsafe behavior that may result in traffic accidents. A project to assess the risk of driving a car in Europe classifies the risk of autonomous driving while taking pharmaceuticals into four categories [11]. The medicine package insert that officially regulates the usage of Japanese medicine classifies the risk into three categories: prohibited, caution, and no cautionary statement required [12]. In a 2013 study that employed national databases in Japan, 43% of outpatients were taking medications that prohibited driving an automobile [29].

In a study of American drivers, many were warned about their medications’ effects on driving, but those who perceived that they were warned were related to socio-population groups, such as medicine type and gender [15]. Studies in the Netherlands have shown that healthcare professionals’ warnings about the effects of driving while on medication are useful for helping patients recognize related risks [30].

In this study, 30% of respondents were taking medications that prohibit driving, of whom 25.7% were not alerted by healthcare professionals. Approximately 16% were given direct warnings about not driving; however, there were many euphemistic or even unclear expressions such as “refrain from calling attention.” Khojah’s study also points out that there is insufficient counseling on driving at community pharmacies [31].

Automobile crashes caused by the effects of medication pose a hazard not only to the patient and family but also to third parties; hence, they can be considered a societal concern. Polypharmacy among middle-aged and elderly patients also affects driving [32].

As Smyth’s research points out, even if people have a high level of knowledge about the medication-related hazards of driving a car, many will adjust their dose or frequency to continue driving [23].

Healthcare professionals are obligated to explain the side effects and promote the proper use of medications, avoid medication-related incidents such as traffic accidents, and explain the medication prescription not only to patients but also to family members. In terms of physical well-being, patients must also ensure medication adherence so that the illness is well controlled. Thus, healthcare professionals must understand the effects of their messages on treatment adherence and driving behavior.

For patients who take medications that significantly affect driving, several factors are important to ensure behaviors to prevent automobile accidents, such as discontinuing driving or consulting a healthcare professional. To achieve this, healthcare professionals must fully understand the social environment of the patient and their family and ensure an environment amenable to advice-seeking by patients. Research into communication between healthcare professionals and elderly drivers has identified that persuasive messaging includes elements of trust, emotion, and context [33] and that it takes time for patients to relinquish driving [34]. A study on safe driving among older drivers, families, and physicians also revealed that very few older drivers discussed safety practices with their families, which was consistent with participants’ living environment [35]. The study also identified a need for risk assessments and family support, as well as adequate communication on the risk factors of driving while on medication [36]. In addition to education and campaigns aimed at understanding the effects of medication on automobile driving, individual counseling is also required.

Medical professionals, such as doctors and pharmacists, should consider patients’ social backgrounds and provide warning messages based on the type of behavior the patients will engage in. Future studies must evaluate how two-way communication between patients and health care professionals can aid drug selection by drivers.

There are several limitations to this study, particularly regarding participant selection bias. First, Internet users are known to have higher education levels, suggesting that they have higher health literacy than non-Internet users. Second, our research is based on self-reporting and some participants may have underreported or misreported. Third, this study was conducted using an internet survey, and data on cognitive function and medical records were not available. The patient’s driving and medication behavior may be affected by cognitive function and disease status. The study could not account for this. Therefore, further research based on cognitive function screening and medical records is needed. Despite these limitations, our findings may contribute to adequate risk communication for patients.

## Conclusions

Our results indicate that healthcare professionals are sometimes unable to fully inform patients about the driving-related risks of medications. To encourage patients taking medications that have a significant impact on driving to either stop driving or consult a healthcare professional, those professionals must first understand the patient’s social environment, such as whether driving is required for work, and then create an environment conducive for advice-seeking intention.

## Data Availability

Data are available from the corresponding author Yasue Fukuda upon reasonable request.
